# Uranium in Drinking Water: Low Dose Acts as Endocrine Mimic

**Published:** 2007-12

**Authors:** Valerie J. Brown

Uranium, the heaviest naturally occurring element, is well known as a radioactive toxicant capable of damaging the kidneys and DNA. A new study has shown for the first time that uranium also acts as an estrogen mimic in mice at concentrations below the U.S. EPA’s safety limit of 30 μg/L in drinking water **[*EHP* 115:1711–1716; Raymond-Whish et al.]**. Other metals, including arsenic, cadmium, lead, and mercury, also are known estrogen mimics.

The researchers manipulated the reproductive status of female mice in several ways. They exposed one group of immature female mice to uranium as they matured, a second group of mature female mice to uranium at environmentally relevant concentrations for 30 days prior to breeding and through gestation, and a third group of female mice to uranium immediately after their ovaries were removed. In a fourth group, they removed the ovaries of female mice, then exposed subgroups of this cohort to either uranium or the synthethic estrogen diethylstilbestrol (DES) alone or in combination with the antiestrogen ICI 182,780. All uranium exposures were via the mice’s drinking water at concentrations of 0.5 μg/L–60.0 mg/L.

Uranium had estrogen-like effects at varying dose ranges throughout the suite of experiments. In the first group, exposure resulted in fewer primary and more secondary ovarian follicles among adult females. In the second group, female pups of exposed dams had significantly fewer small primary ovarian follicles. The researchers conjecture that this primary-to-secondary follicle ratio may lead to fewer ovulated eggs and early-onset menopause. In the ovariectomized mice, the researchers found higher uterine weights and accelerated vaginal opening (indicators of earlier puberty onset). In addition, estrogenic activity was blocked in the mice exposed to ICI 182,780 after DES or uranium exposure.

The current study is of immediate relevance to the Navajo Nation of Arizona and New Mexico, where many rural Navajo water supplies currently contain uranium at concentrations exceeding the U.S. EPA standard. The uranium boom of the 1950s and 1960s left thousands of abandoned mine sites and derelict milling operations on Navajo lands. Uranium mining has been banned there, but there are active efforts to revive uranium mining in the Navajo town of Crownpoint, New Mexico. The findings may also soon apply to other populations living amid the uranium boom now under way in central Colorado, Canada, Australia, and elsewhere.

## Figures and Tables

**Figure:**
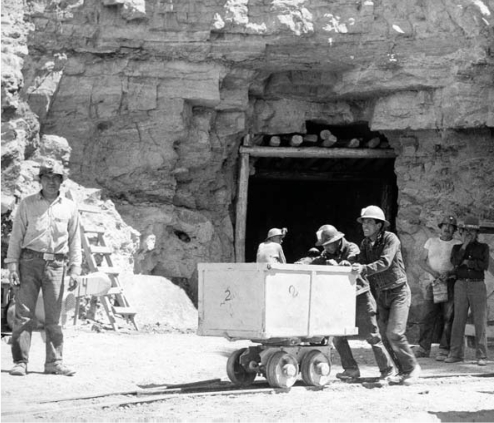
Mine memory Navajo miners work the Kerr-McGee uranium mine, 7 May 1953. Today, uranium from unremediated abandoned mines contaminates nearby water supplies.

